# Supplementary material for: Dominant role of DNA methylation over H3K9me3 for IAP silencing in endoderm
Source: Nat Commun. 2022 Sep 19;13:5447. doi: 10.1038/s41467-022-32978-7 (PMC9485127; doi:10.1038/s41467-022-32978-7)
Supplement: Supplementary file 1 — Supplementary Information [file 41467_2022_32978_MOESM1_ESM.pdf]

Supplementary Information

Cell lines used and generated in this study

Cell line	ID	type	origin	generation
Setdb1$^{flox/+}$; Sox17-2A-iCre; EGFP-reporter	B70-1	feeder-dependent mouse ES cells	this study	derived from blastocysts
Setdb1$^{flox/flox}$; Sox17-2A-iCre; EGFP-reporter	B63-4	feeder-dependent mouse ES cells	this study	derived from blastocysts
J1 wildtype ES cells	J1 wt	feeder-independent mouse ES cells	Leonhardt lab	-
Dnmt1 ko ES cells	D1 ko	feeder-independent mouse ES cells	Leonhardt lab	-
293T cells	293T	human embryonic kidney cells	ATCC	-
Suv39h dko ES cells	dn57	feeder-independent mouse ES cells	(Peters et al., 2001)	-
wild type control ES cells	wt26	feeder-independent mouse ES cells	(Peters et al., 2001)	-

Antibodies

**Epitope**	**Host**	**Type**	**Company**	**Product Number**
Setdb1	Rabbit	polyclonal	Santa Cruz	Anti-Setdb1 (sc66884-X)
a-Tubulin	Mouse	monoclonal	Sigma Aldrich	anti-a-Tubulin (clone B-5-1-2)
GFP	Chicken	polyclonal	Aves	Anti-GFP (GFP1020)

AFP	Mouse	polyclonal	R&D	Anti-AFP (af5369)
Foxa2	Rabbit	polyclonal	Abcam	Anti-Foxa2 (ab40874)
Sox17	Goat	polyclonal	Neuromics	Anti-Sox17 (gt15094)
IAP-GAG	Rabbit	polyclonal	Bryan Cullen lab	Anti-IAP-GAG
H3K9me3	Rabbit	polyclonal	Active Motif	Anti-H3K9me3 (39161)

qPCR Primers

target	ID	direction	sequence (5' to 3' direction)	use	taken from reference
Gapdh	GS276.1	fw	TCAAGAAGGTGGTGAAGCAG	used as reference	
	GS276.2	rw	GTTGAAGTCGCAGGAGACAA		
Hprt	GS279.1	fw	ATGAGCGCAAGTTGAATCTG	used as reference	
	GS279.2	rw	CAGATGGCCACAGGACTAGA		
Setdb1-exon4	GS958	fw	AGCAGAACTCCAAAAGACCAGA AGC	Fig S2D, Fig S3B	
	GS959	rw	TCTTGCCCAGAATCCGCATG		
Sox17	GS2755	fw	GCTAGGCAAGTCTTGGAAGG	Fig S3B	
	GS2756	rw	CTTGTAGTTGGGGTGGTCCT		
IAP-global	GS2512	fw	CGGGTCGCGGTAATAAAGGT	Fig S3B	(Sadic et al. 2015)
	GS2513	rw	ACTCTCGTTCCCCAGCTGAA		

Primers for bisulfite-PCR-analysis

target	ID	direction	sequence (5' to 3' direction)	use	taken from

					reference
IAP-Gag	GS4289	fw	AGGTTAGTTTGTTGATTGGTTTTAG	Fig 4C	(Sadic et al. 2015)
	GS4290	rw	AATCAACAAAATAAACTCCCTAACC		
IAP-LTR	GS4261	fw	GGTTTTGGAATGAGGGATTTT	Fig 4C	(Rowe et al. 2013)
	GS4262	rw	CTCTACTCCATATACTCTACCTTC		
LINE-1	GS4291	fw	GTTAGAGGATTTGATAGTTTTTGGAATAGG	Fig S4B	(Tommasi et al. 2012)
	GS4292	rw	CCAAAACAAAACCTTTCTCAAACACTATAT		

ChIP-qPCR Primers

target	ID	direction	sequence (5' to 3' direction)	use	taken from reference
Gapdh	GS485	fw	CCATCCCACGGCTCTGCAC	Fig 5B	
	GS486	rw	GCAAGGCTTCCGTGCTCTCG		
Polrmt	GS1650	fw	TCAGCAAACTCCAATAGCGCAC	Fig 5B	
	GS1651	rw	TTGCCGCACAACATGGACTT		
H19	GS928	fw	AGCTTTGAGTACCCCAGGTTCA	Fig 5B	
	GS929	rw	GCCTCTGCTTTTATGGCTATGG		
IAP-LTR	GS3804	fw	GCTCCTGAAGATGTAAGCAATAAAG	Fig 5B	(Maksakova et al. 2013)
	GS3805	rw	CTTCCTTGCGCCAGTCCCGAG		
IAP-global	GS2512	fw	CGGGTCGCGGTAATAAAGGT	Fig 5B	(Sadic et al. 2015)
	GS2513	rw	ACTCTCGTTCCCCAGCTGAA		

IAP-GAG	GS3908	fw	CACGCTCCGGTAGAATACTTACAAAT	Fig 5B	(Sharif et al. 2016)
	GS3909	rw	CCTGTCTAACTGCACCAAGGTAAAAT		

Plasmids

ID	plasmid name	Inserted fragments	origin	comment and usage
GS183	psPAX2	-	Didier Trono (Addgene: 12260)	Used for lentiviral packaging
GS811	pLPeco-env	-	(Dambacher et al, 2012)	Used for lentiviral packaging
GS1582	pLenti6/EF1a-GATA6-IRES-Puro	GATA6	(Sadic et al. 2015)	Stable overexpression

a

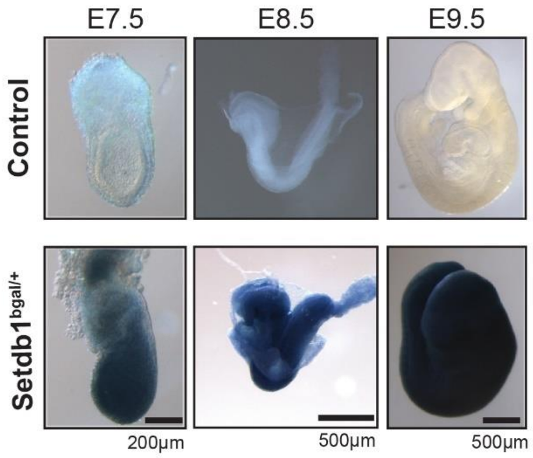

b

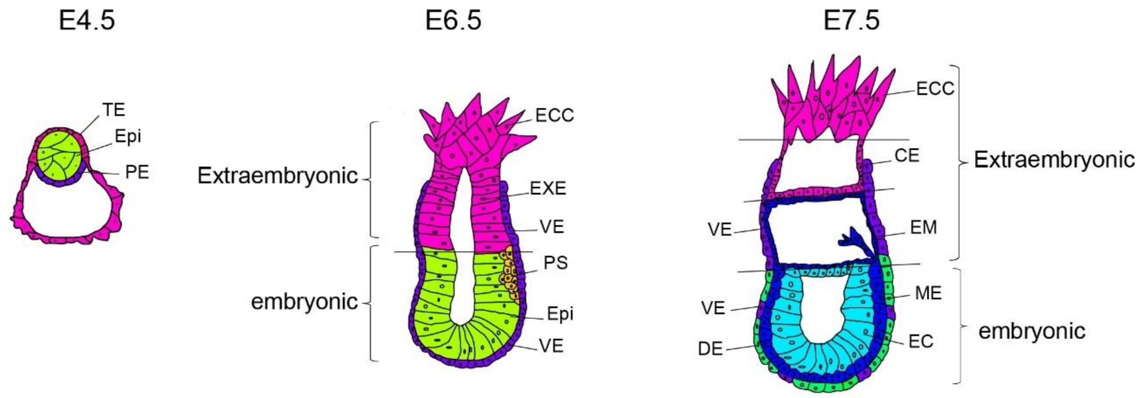

TE: Trophectoderm
Epi: Epiblast
PE: Primitive Endoderm
EXE: Extraembryonic Ectoderm
ECC: Ectoplacental Cone
PS: Primitive Streak
VE: Visceral endoderm

CE: Chorionic ectoderm
EM: Extraembryonic mesoderm
ME: Mesoderm
DE: Definitive endoderm
EC: Ectoderm

At E6.5, extraembryonic and embryonic visceral endoderm cells are derived from primitive endoderm cells. Meanwhile, cells start to migrate through the primitve streak region to form definitive endoderm and mesoderm. At E7.5, the visceral endoderm cells become displaced by the intercalation of the definitive endoderm cells.

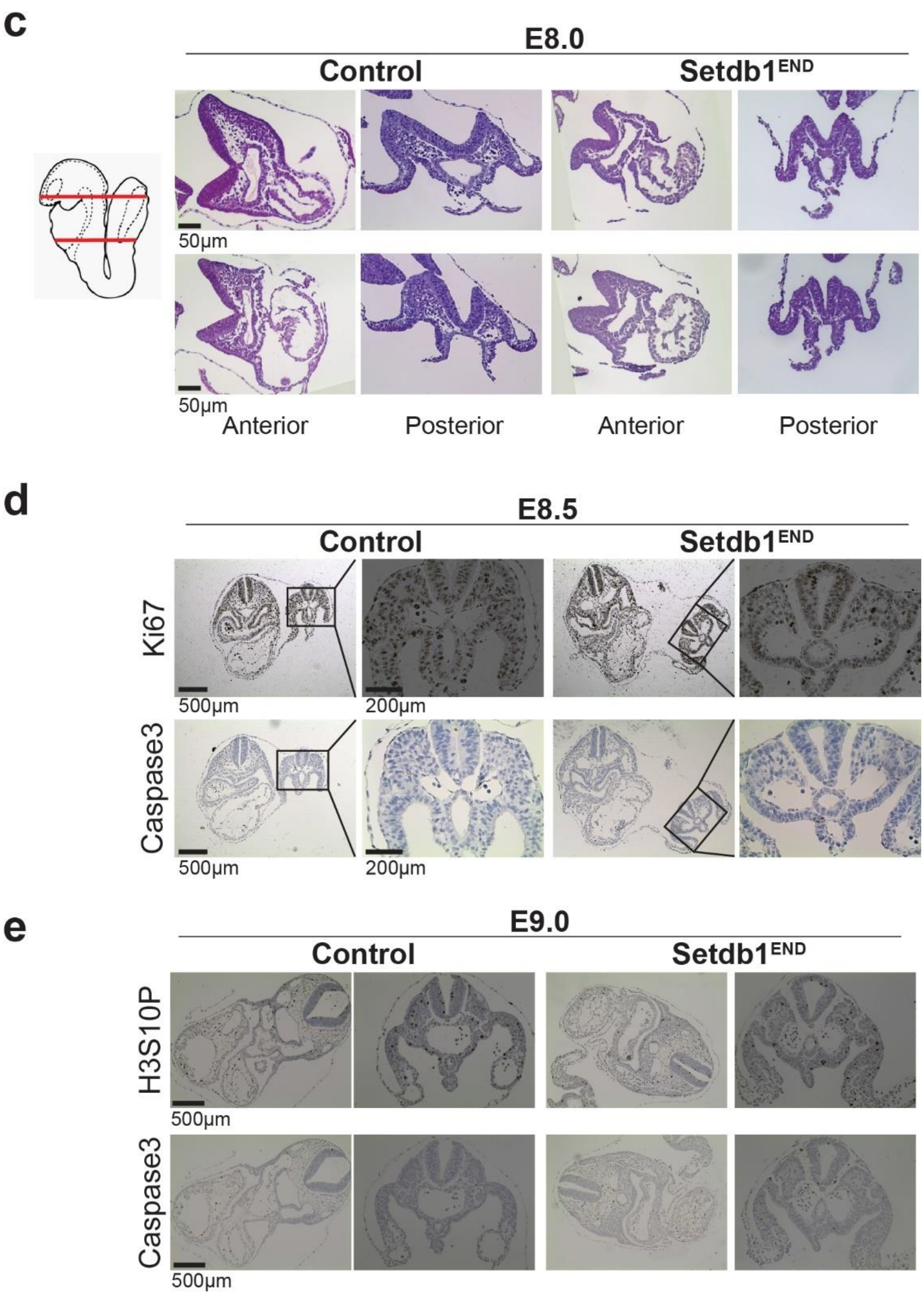

Supplementary Figure 1.

(a) *Setdb1* is ubiquitously expressed in early embryos. Activity of β-galactosidase was measured in control (no staining) and *Setdb1*$^{\beta gal}$ embryos from E7.5 to E9.5 (anterior to the left). Representative images from n=3 per genotype and stage.

(b) Schematic of early embryonic development and lineage differentiation. Deletion of *Setdb1* occurs in visceral and definitive endoderm cells.

(c) Hematoxylin/Eosin staining of transverse sections of E8.0 control and *Setdb1*^{END} embryos. The approximate positions of the sections are indicated in the schematic. No obvious structural aberrations could be detected. Representative images from n=3 per genotype.

(d) Immunohistochemistry analysis of Caspase3 (apoptosis marker) and Ki67 (proliferation marker) of transverse sections from E8.5 control and *Setdb1*^{END} embryos. Rectangles mark the posterior parts shown in the magnified views. Representative images from n=3 per genotype.

(e) Immunohistochemistry analysis of Caspase3 (apoptosis marker) and H3S10P (mitosis marker) of transverse sections from E9.0 control and *Setdb1*^{END} embryos. Rectangles mark the posterior parts shown in the magnified views. Representative images from n=3 per genotype.

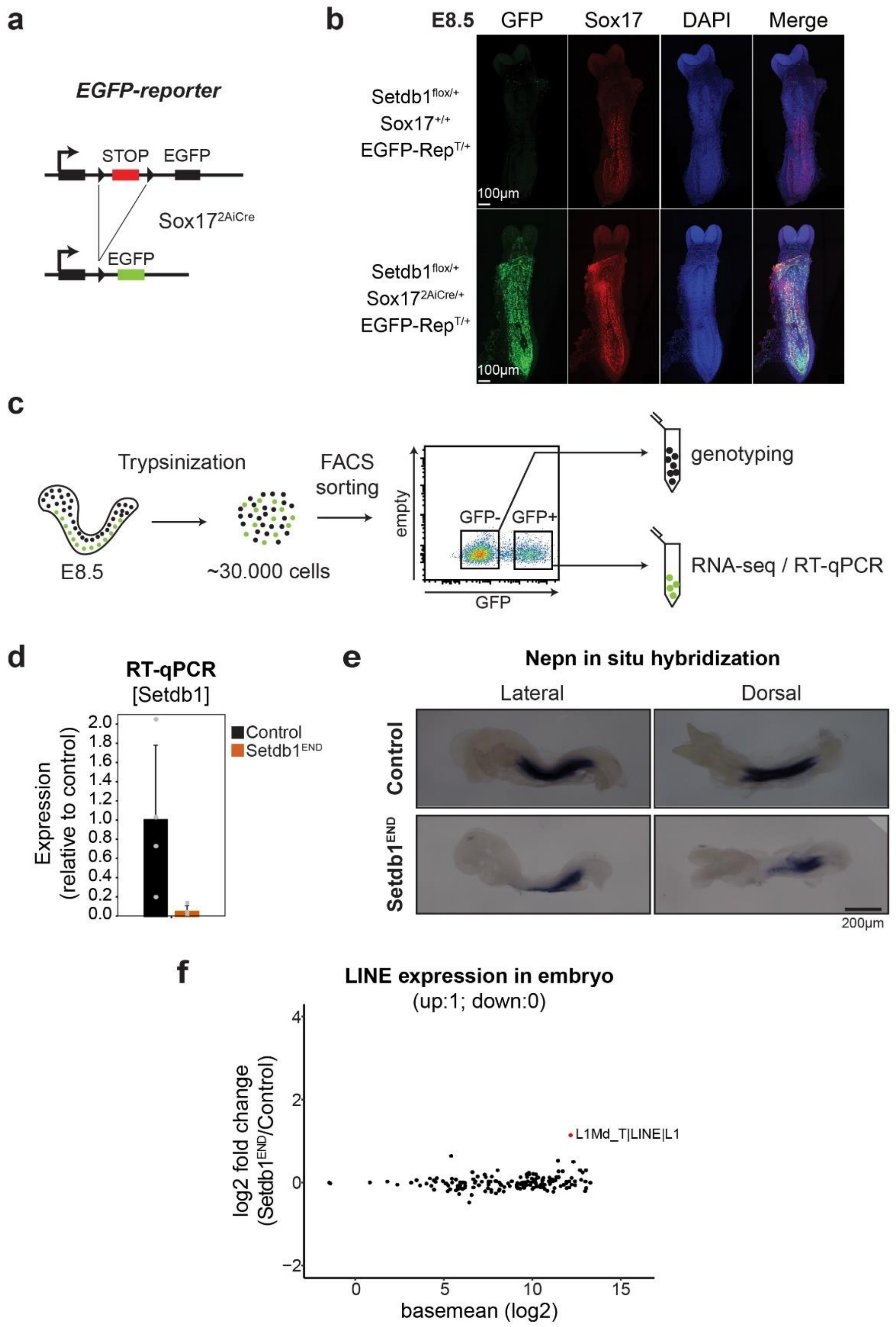

Supplementary Figure 2.

(a) Schematic of the *EGFP Cre* reporter allele. A floxed STOP cassette precedes the EGFP coding region. Deletion of this STOP cassette by *Sox17*$^{2A\text{-}iCre}$ results in EGFP expression in cells with *Cre* activity.

(b) Whole mount staining of EGFP (marks activity of the *Cre* reporter) and *Sox17* (marks endoderm cells) in E8.5 control embryos with (lower panel) or without (upper panel) *Sox17*$^{2A\text{-}}$

^{iCre} allele. EGFP reporter activity strictly depends on presence of *Sox17*$^{2A\text{-}iCre}$ and overlaps with the *Sox17* expression domain. Representative images from n=3 per genotype.

(c) Schematic of the FACS sorting strategy for endoderm cells from E8.5 embryos based on EGFP reporter expression.

(d) Expression analysis of *Setdb1* by RT-qPCR in control and *Setdb1*^{END} embryonic endoderm cells relative to control. Data are presented as mean values +/- SD from replicate experiments (n=4). Source data are provided as a Source Data file.

(e) In situ hybridization analysis for *Nepn* in control and *Setdb1*^{END} embryos. Reduced *Nepn* expression was detected in *Setdb1*^{END} embryos. Representative images from n=3 control and n=2 mutant embryos.

(f) Dot plot showing basemean expression vs. log2-fold change of LINEs in embryonic endoderm cells. LINEs with significantly changed expression (Wald test with Benjamini-Hochberg correction, adjusted p-value < 0.01, fold change > 2; n=3 for each condition) are colored (red = increased expression in *Setdb1*^{END} cells, blue = reduced expression in *Setdb1*^{END} cells). Selected LINEs are labeled.

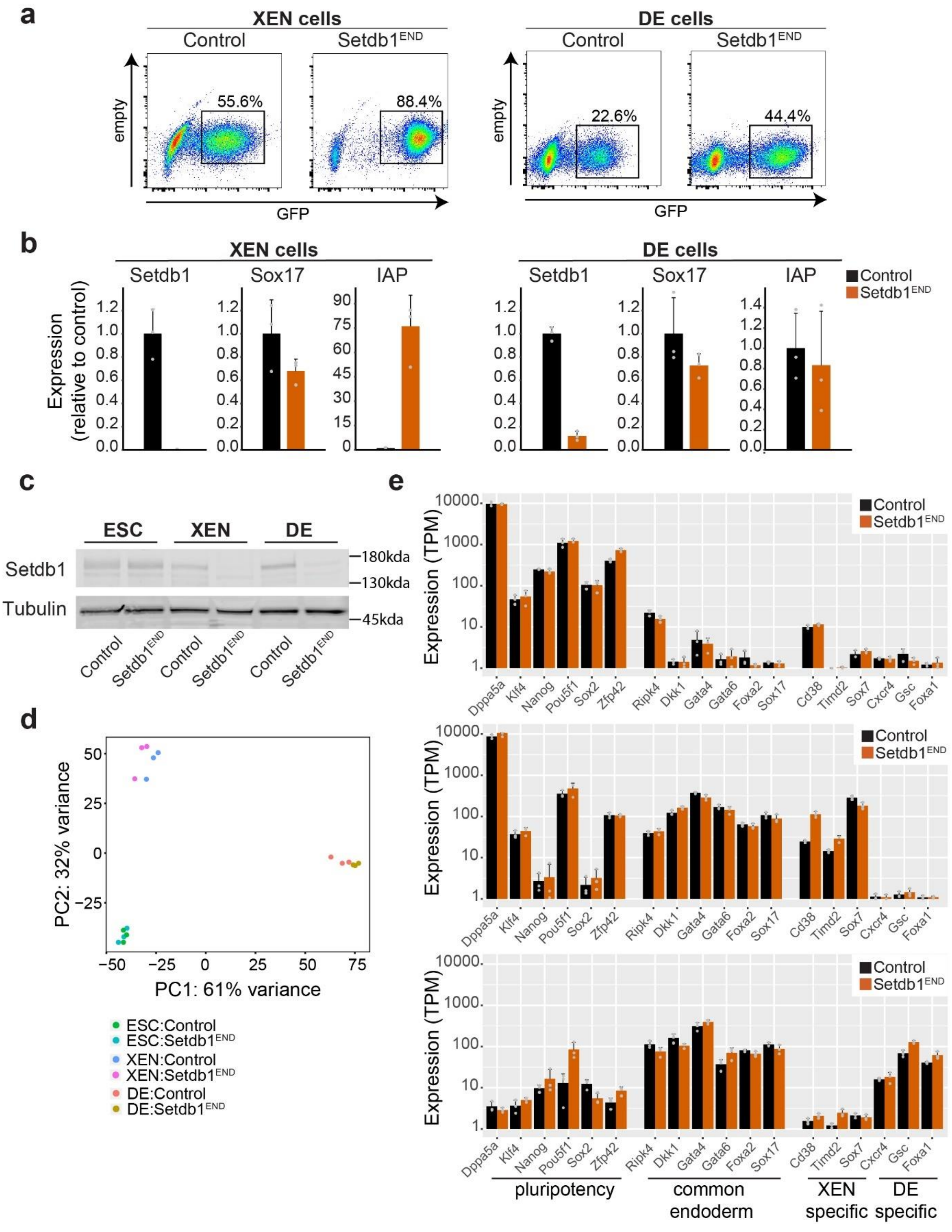

a
XEN cells
DE cells
Control
Setdb1END
empty
GFP
55.6%
88.4%
22.6%
44.4%
b
XEN cells
DE cells
Setdb1
Sox17
IAP
Control
Setdb1END
Expression (relative to control)
c
ESC
XEN
DE
Setdb1
Tubulin
180kda
130kda
45kda
Control
Setdb1END
d
PC1: 61% variance
PC2: 32% variance
ESC:Control
ESC:Setdb1END
XEN:Control
XEN:Setdb1END
DE:Control
DE:Setdb1END
e
Expression (TPM)
Control
Setdb1END
Dppa5a
Klf4
Nanog
Pou5f1
Sox2
Zfp42
Ripk4
Dkk1
Gata4
Gata6
Foxa2
Sox17
Cd38
Timd2
Sox7
Cxcr4
Gsc
Foxa1
pluripotency
common endoderm
XEN specific
DE specific

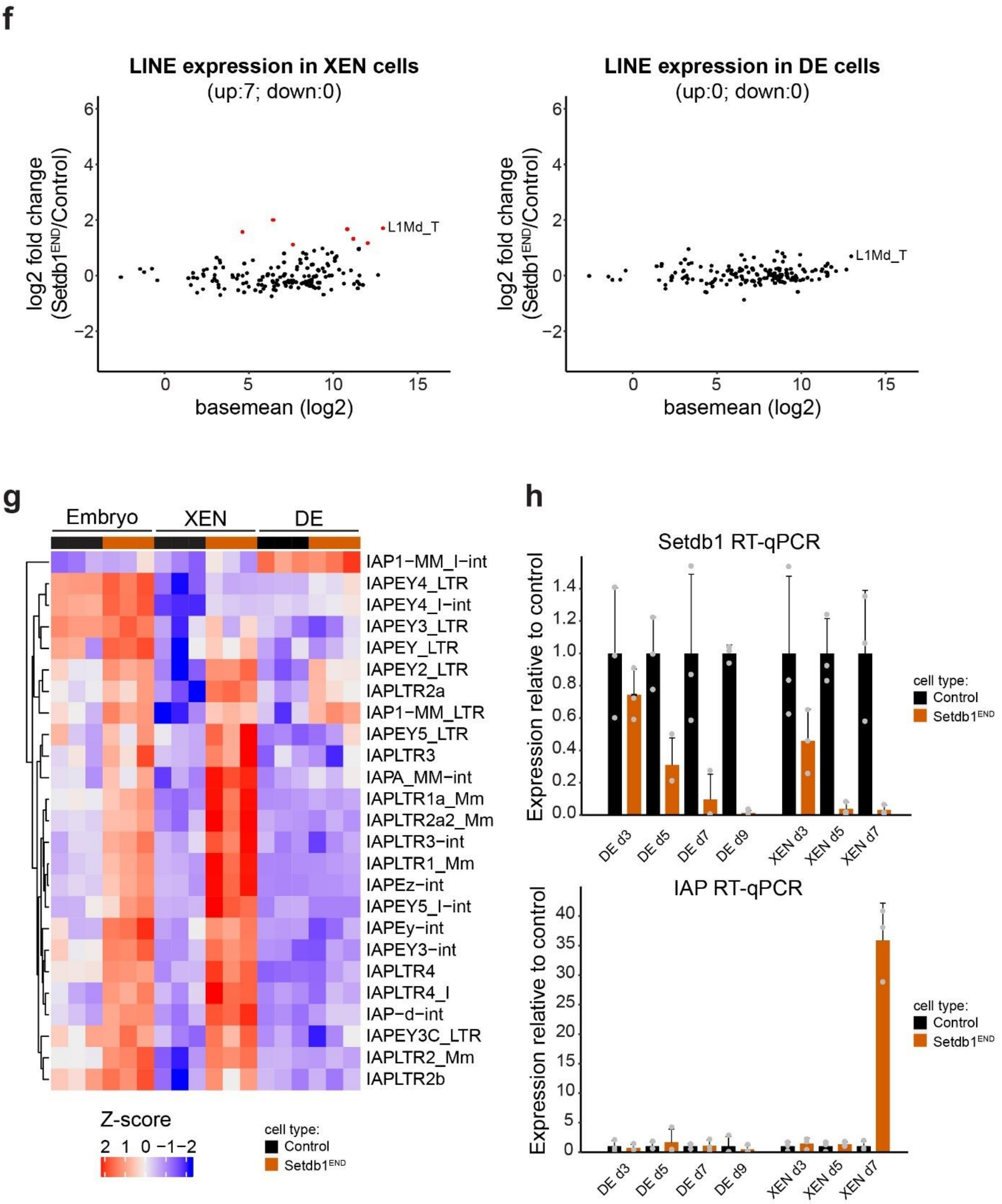

Supplementary Figure 3.

(a) Representative FACS plots of *in vitro* differentiated ESCs. EGFP expression corresponds to activation of the EGFP reporter by *Sox17*$^{2A\text{-}iCre}$ upon endoderm commitment. EGFP positive XEN od DE cells were FACS sorted for subsequent analyses.

(b) Expression of *Setdb1*, *Sox17* and *IAP* elements was detected by RT-qPCR in *in vitro* differentiated control and *Setdb1*^{END} XEN and DE cells relative to control cells. Data are

presented as mean values +/- SD from replicate experiments (n=3). Source data are provided as a Source Data file.

(c) Western blot detection of SETDB1 protein expression in control and *Setdb1*^{END} ESCs, XEN and DE cells. Tubulin serves as loading control. Source data are provided as a Source Data file.

(d) PCA analysis of RNA-seq data from control and *Setdb1*^{END} ESCs, XEN and DE cells. Each dot represents one individual replicate.

(e) Average expression levels of genes detected by RNA-seq for pluripotency, extra-embryonic endoderm and definitive endoderm markers in ESCs, XEN and DE cells. TPM: Transcripts Per Kilobase Million. Data are presented as mean values +/- SD from replicate experiments (n=3).

(f) Dot plot showing basemean expression vs. log2 fold change of LINEs in *in vitro* differentiated control and *Setdb1*^{END} XEN and DE cells. LINEs with significantly changed expression (Wald test with Benjamini-Hochberg correction, adjusted p-value < 0.01, log2foldchange > 1; n=3 for each condition) are colored (red = increased expression in *Setdb1*^{END} cells, blue = reduced expression in *Setdb1*^{END} cells). Selected LINEs are labeled.

(g) Heatmap showing expression of *IAP* family members in control and *Setdb1*^{END} embryonic endoderm and *in vitro* differentiated XEN and DE cells. Z score indicates expression differences (red = high, blue= low). Hierarchical clustering was performed using complete agglomeration method and a Euclidean distance metric (n=3 for each condition).

(h) Time-course experiment for *Setdb1* deletion in *Setdb1*^{END} DE and XEN cells. Expression analysis of *Setdb1* and *IAPEz* by RT-qPCR in control and *Setdb1*^{END} DE cells relative to control. Data are presented as mean values +/- SD from replicate experiments (n=3). Source data are provided as a Source Data file.

Control and *Setdb1*^{END} ES cells were *in vitro* differentiated. RNA was isolated at day 3,5,7 (DE and XEN) and 9 (DE) days of differentiation. RT-qPCR analysis for *Setdb1* and *IAPEz* expression revealed that XEN cells display strong *IAPEz* de-repression at day 7 of differentiation when *Setdb1* was strongly deleted for 2 days. In contrast, DE cells did not display *IAPEz* expression even at day 9 of differentiation.

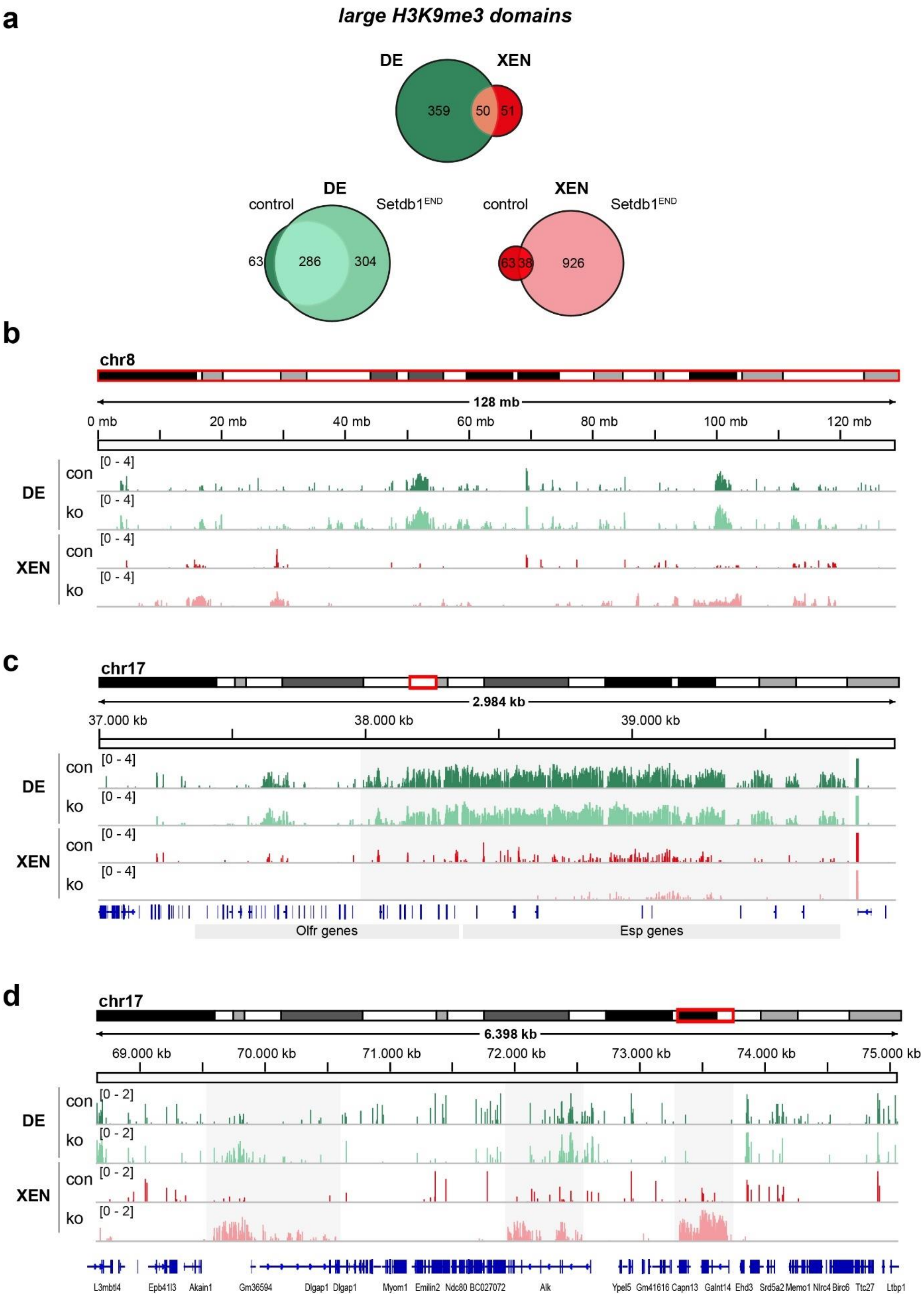

Supplementary Figure 4.

(a) Megabase-size H3K9me3 domains are detected predominantly in DE cells. ChromstaR was used to detect large stage-specific H3K9me3 domains. In DE cells, prominent domains

could be identified, which only partially overlapped with XEN cell, where large H3K9me3 domains were less apparent. In *Setbd1*^{END} DE cells these domains are largely unaltered. Appearance of new H3K9me3 domains was observed in *Setdb1*^{END} XEN cells.

(b) Visualization of megabase-sized H3K9me3 domains on chromosome 8. H3K9me3 tracks for control and *Setdb1*^{END} DE and XEN cells are shown.

(c) Example of a DE-specific H3K9me3 domain (gray box) on chromosome 17 covering *Olfr* and *Esp* genes which are not expressed in DE or XEN cells.

(d) Example of *Setdb1*^{END}-specific H3K9me3 domains in XEN cells (gray boxes).

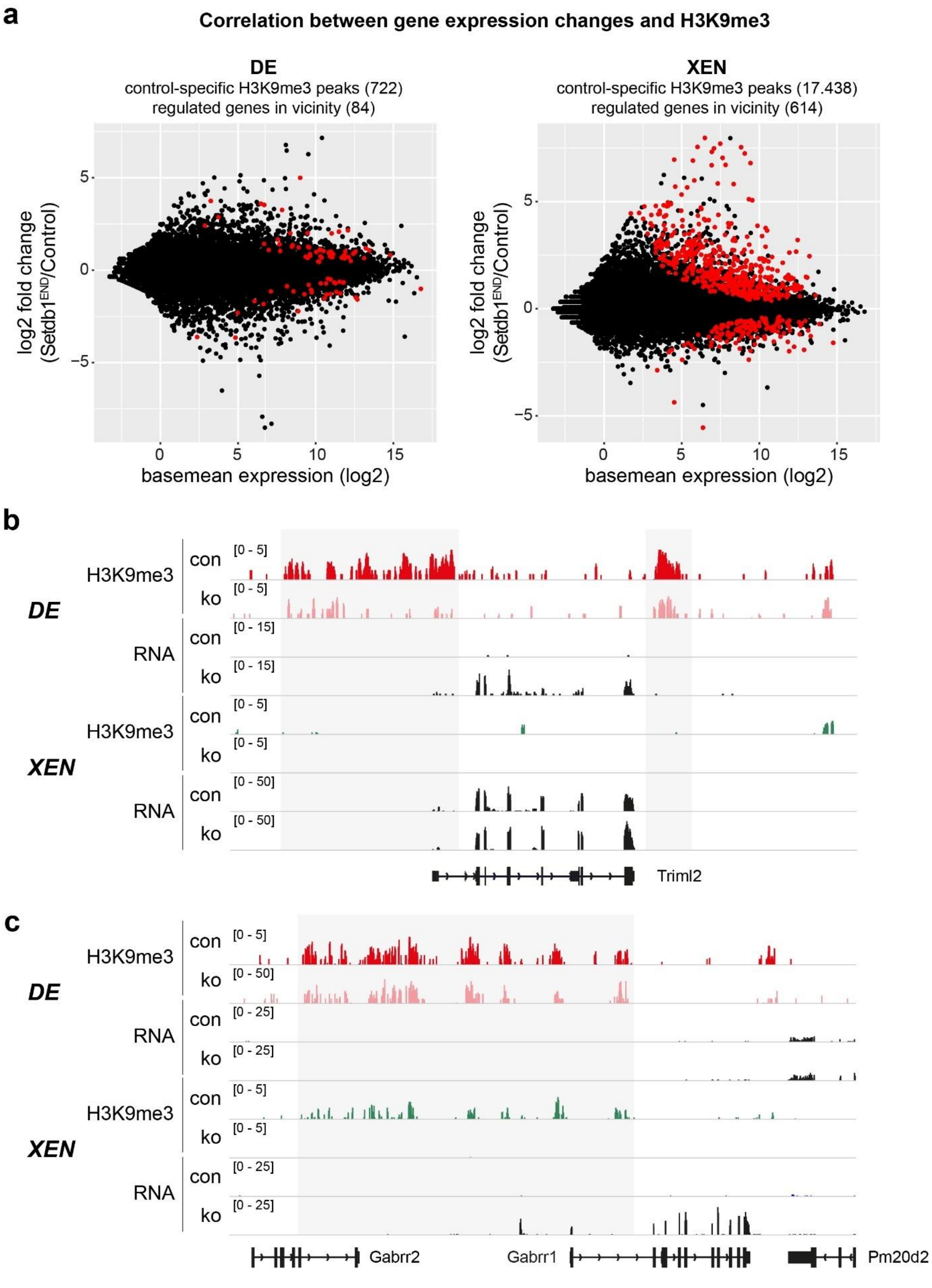

Supplementary Figure 5.

(a) Correlation between gene expression changes and changes in H3K9me3 in *Setdb1*^{END} DE and XEN cells. H3K9me3 peaks which lose the modification in *Setdb1*^{END} cells were detected

with chromstaR. Regulated genes in vicinity of these peaks (< 100 kb to TSS) were identified. Dot plot showing basemean expression vs. log2-fold change of protein coding genes in *in vitro* differentiated control vs. *Setdb1*^{END} XEN and DE cells. Genes with significantly changed expression (Wald test with Benjamini-Hochberg correction, adjusted p-value < 0.01; n=3 for each condition) and presence of a H3K9me3 peak with lost signal in *Setdb1*^{END} cells are colored in red.

(b) Example for a gene with H3K9me3-dependent expression change in DE cells. H3K9me3 was lost in the promoter region and a region downstream of *Triml2* (gray boxes). *Triml2* is not expressed in DE cells but becomes active upon loss of H3K9me3 in *Setdb1*^{END} DE cells.

(c) Example for a gene with H3K9me3-dependent expression change in XEN cells. H3K9me3 was lost in several regions upstream of *Gabrr1* specifically in *Setdb*^{END} XEN cells. *Gabrr1* is not expressed in XEN cells but becomes active upon loss of H3K9me3 in *Setdb1*^{END} XEN cells.

a

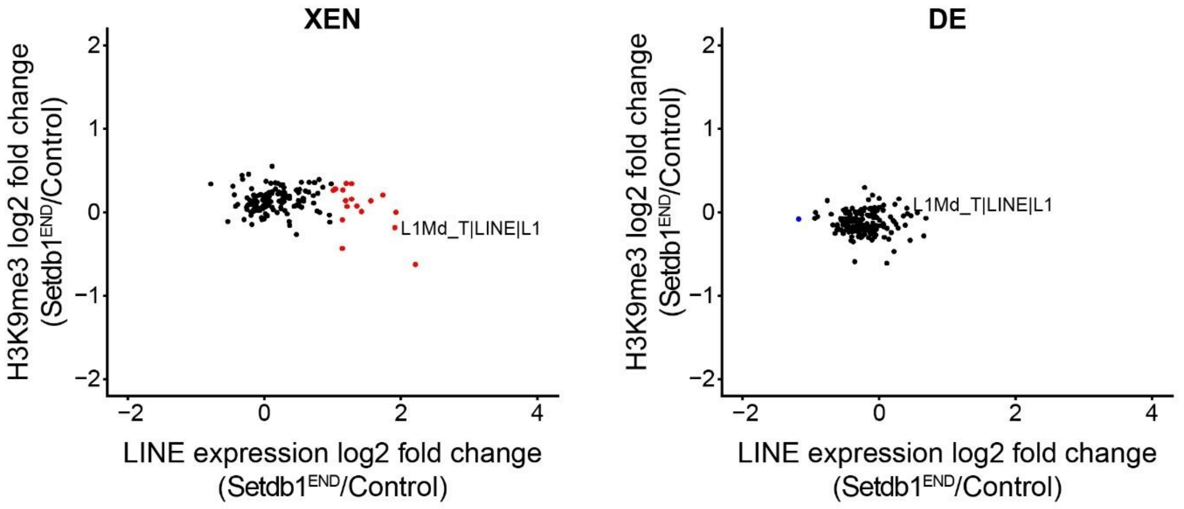

b

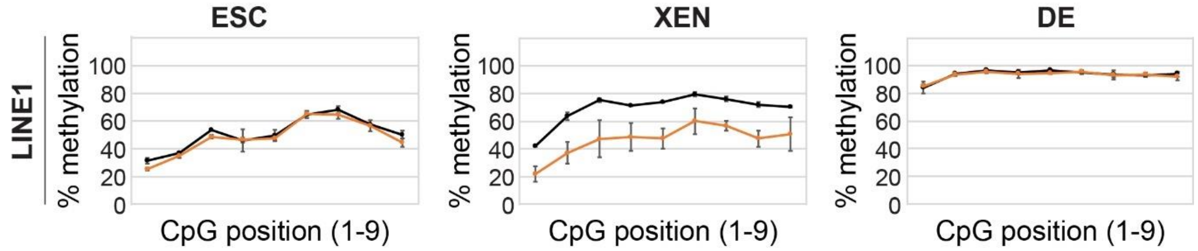

Supplementary Figure 6.

(a) Dot plot showing expression vs. H3K9me3 changes between control and *Setdb1*^{END} XEN and DE cells. LINEs with significantly changed expression (fold change > 2; n=3 for each condition) are colored (red = increased expression in *Setdb1*^{END} cells). Selected LINEs are labeled.

(b) Bisulfite-PCR analysis for DNA methylation in LINE1 elements. Plots display the percentages of DNA methylation in individual CpG positions of LINE1 PCR fragments. DNA methylation analysis was performed in control and *Setdb1*^{END} ESCs, XEN and DE cells Data are presented as mean values +/- SD from replicate experiments (n=2; 500 sequences each). Source data are provided as a Source Data file.

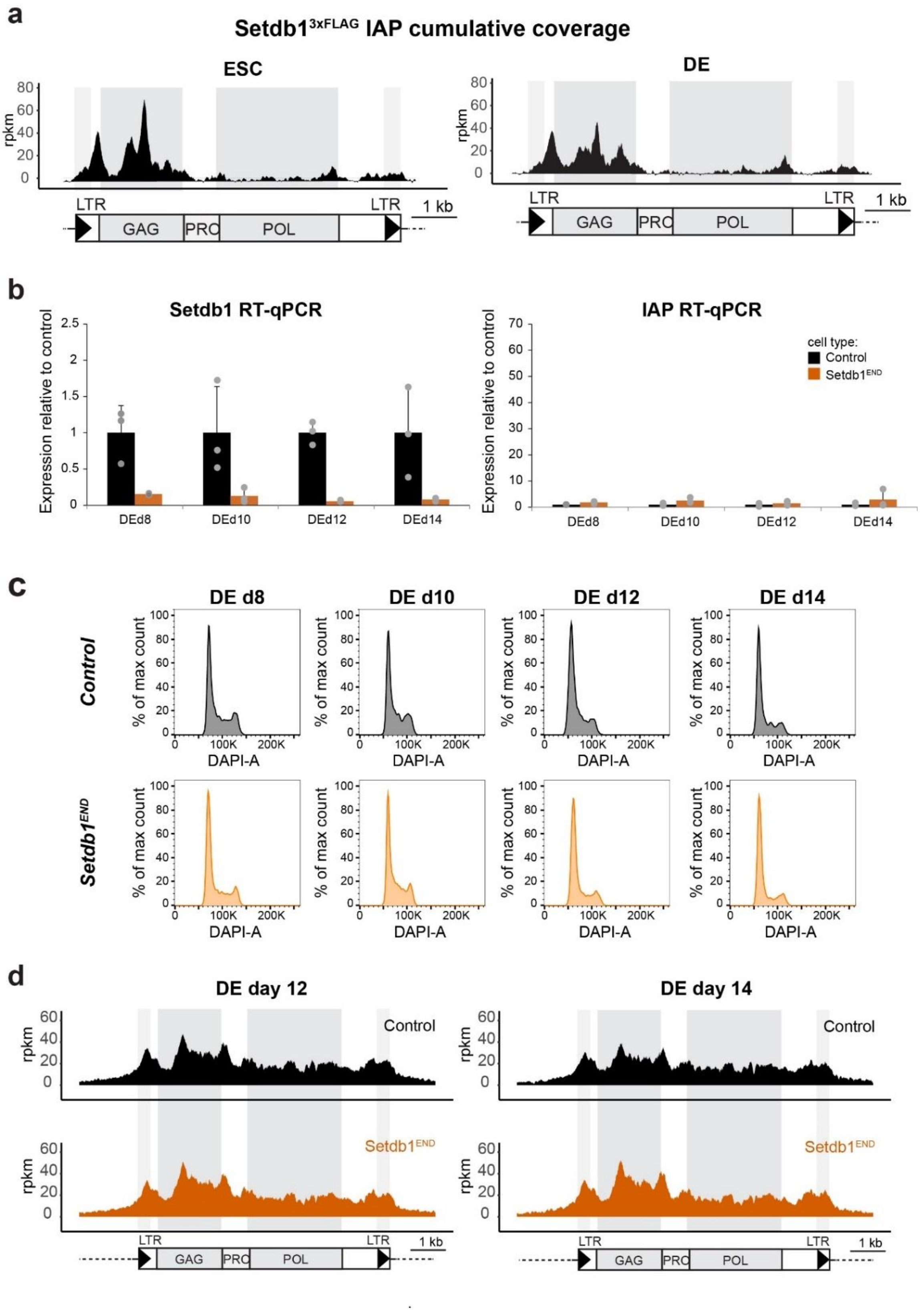

Supplementary Figure 7.

(a) Cumulative Setdb1-3xFLAG ChIP-seq coverage across *IAP* elements in wild type ES and DE cells. The structure of *IAP* elements is shown schematically. (rpkm = reads per kilobase per million of reads).

(b) Extended time-course experiment for *Setdb1* deletion in *Setdb1^{END}* DE cells. Expression analysis of *Setdb1* and *IAPEz* by RT-qPCR in control and *Setdb1^{END}* DE cells relative to control. Data are presented as mean values +/- SD from replicate experiments (n=3).

Control and *Setdb1^{END}* ES cells were *in vitro* differentiated. RNA was isolated at days 8,10,12 and 14 of differentiation. DE cells did not display noticeable *IAPEz* expression within this time-course. Source data are provided as a Source Data file.

(c) Cell cycle analysis of DE cells during the extended differentiation time-course. Control and *Setdb1^{END}* DE cells at days 8, 10, 12 and 14 of differentiation were stained with Hoechst to stain DNA and analyzed by FACS for DNA content representing G1, S and G2/M populations. Cell cycle profiles representative of 3 replicates are shown.

(d) Cumulative H3K9me3 ChIP-seq coverage across IAP elements in control and *Setdb1^{END}* DE cells at days 12 and 14 of differentiation. The structure of *IAP* elements is shown schematically. (rpkm = reads per kilobase per million of reads).

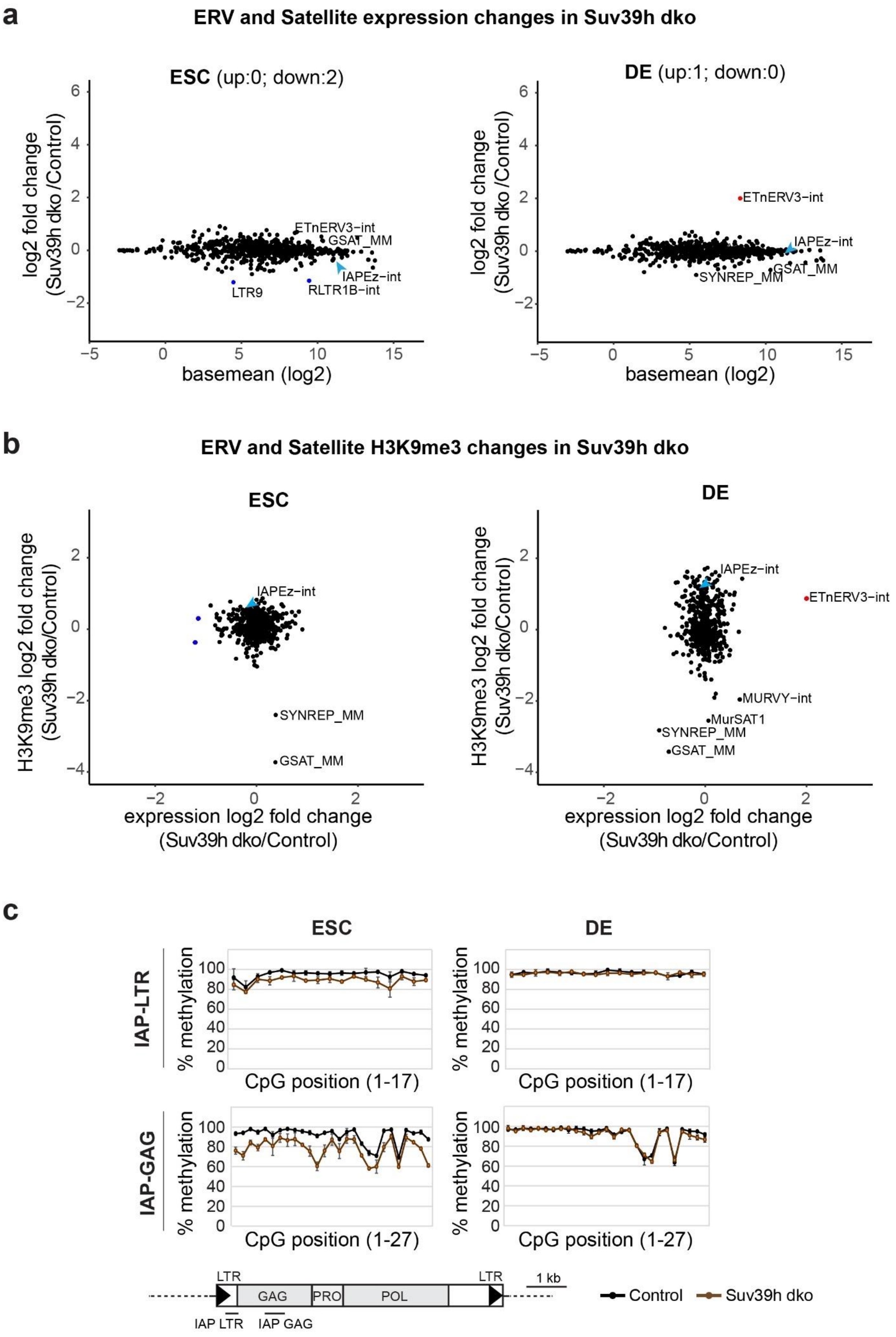
a
ERV and Satellite expression changes in Suv39h dko
ESC (up:0; down:2)
DE (up:1; down:0)
log2 fold change (Suv39h dko /Control)
basemean (log2)
ETnERV3-int
GSAT_MM
IAPEz-int
LTR9
RLTR1B-int
SYNREP_MM
b
ERV and Satellite H3K9me3 changes in Suv39h dko
ESC
DE
H3K9me3 log2 fold change (Suv39h dko/Control)
expression log2 fold change (Suv39h dko/Control)
MURVY-int
MurSAT1
c
IAP-LTR
IAP-GAG
% methylation
CpG position (1-17)
CpG position (1-27)
LTR
GAG
PRO
POL
1 kb
IAP LTR
IAP GAG
Control
Suv39h dko

d

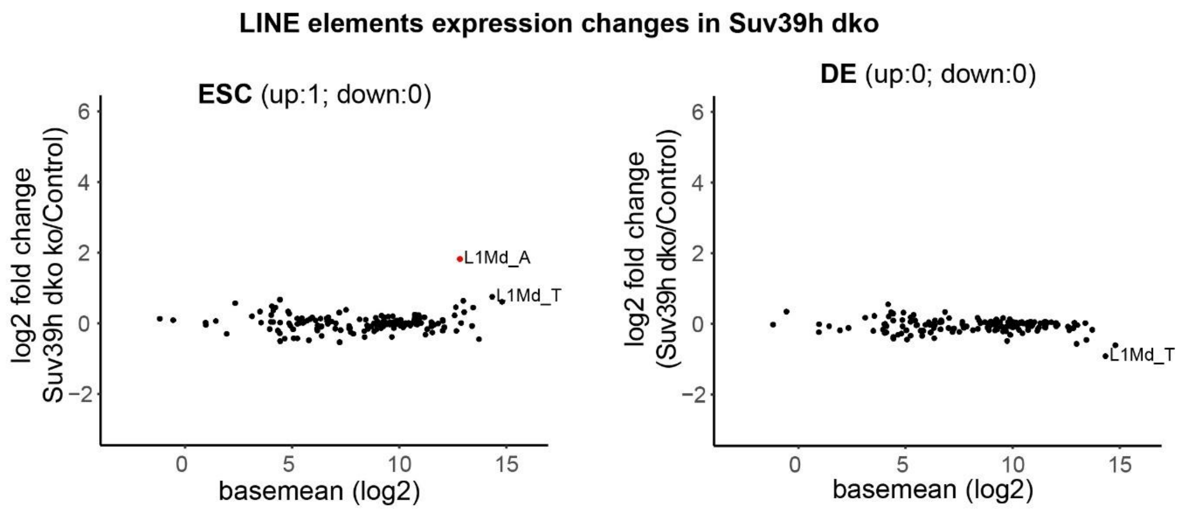

e

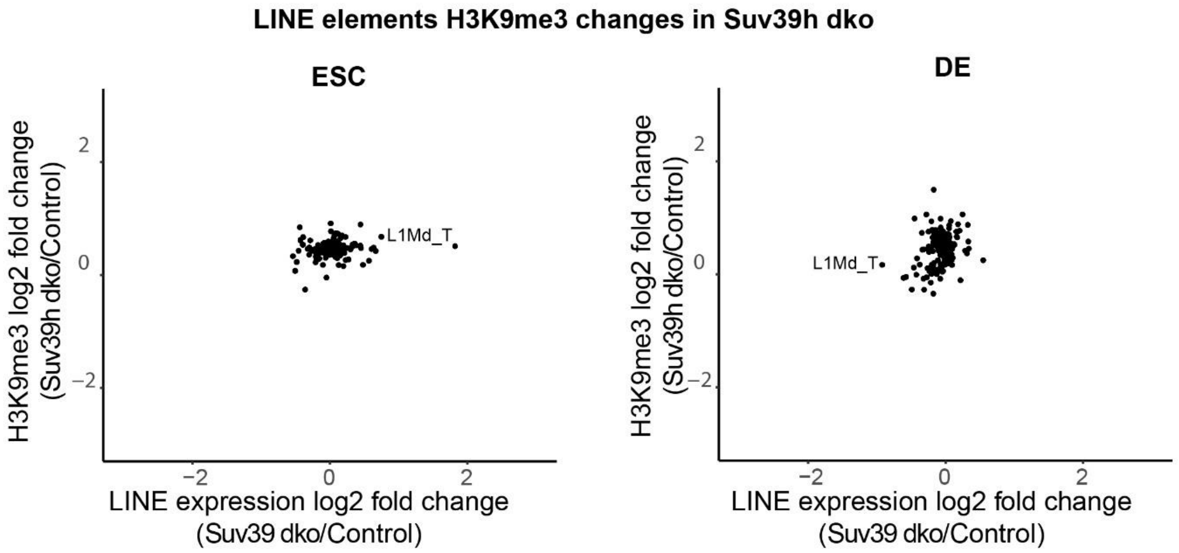

f

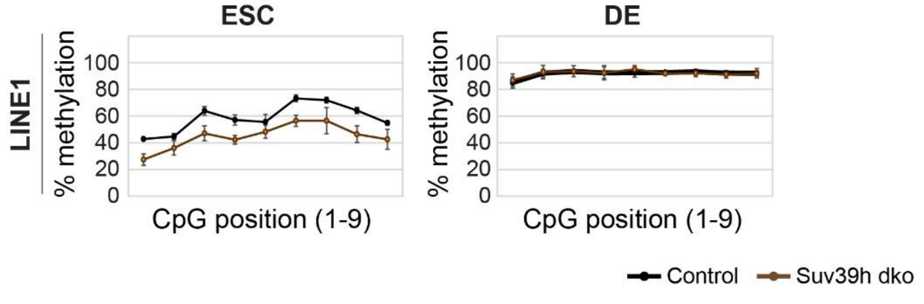

Supplementary Figure 8.

(a) Dot plot showing basemean expression vs. log2-fold change of ERV and satellite repeats families in *in vitro* differentiated control vs. *Suv39h* dko ES and DE cells. Families with significantly changed expression (Wald test with Benjamini-Hochberg correction, adjusted p-

value < 0.01; n=3 for each condition) are colored (red = increased expression in *Suv39h* dko cells, blue = reduced expression in *Suv39h* dko cells). Selected families are labeled.

(b) Dot plot showing basemean expression vs. H3K9me3 change of ERV and satellite repeats families in ESCs and *in vitro* differentiated wild type vs. *Suv39h* dko ES and DE cells. Families with significantly changed expression (Wald test with Benjamini-Hochberg correction, adjusted p-value < 0.01, fold change > 2; n=2 for each condition) are colored (red = increased expression in *Suv39h* dko cells, blue = reduced expression in *Suv39h* dko cells). Selected families are labeled.

(c) Bisulfite-PCR analysis for DNA methylation in *IAP-LTR* and *IAP-GAG* regions. Positions of the PCR products are indicated in the schematic. Plots display the percentages of DNA methylation in individual CpG positions of *IAP-LTR* and *IAP-GAG* PCR fragments. DNA methylation analysis was performed in control and *Suv39h* dko ESCs and DE cells. Data are presented as mean values +/- SD from replicate experiments (n=2; 500 sequences each). Source data are provided as a Source Data file.

(d) Dot plot showing basemean expression vs. H3K9me3 change of LINE families in *in vitro* differentiated control vs. *Suv39h* dko ES and DE cells. LINE families with significantly changed expression (Wald test with Benjamini-Hochberg correction, adjusted p-value < 0.01; n=3 for each condition) are colored (red = increased expression in *Suv39h* dko cells, blue = reduced expression in *Suv39h* dko cells). Selected LINE families are labeled.

(e) Dot plot showing basemean expression vs. log2-fold change of LINE families in ESCs and *in vitro* differentiated wild type vs. *Suv39h* dko XEN and DE cells. LINE families with significantly changed expression (Wald test with Benjamini-Hochberg correction, adjusted p-value < 0.01, fold change > 2; n=2 for each condition) are colored (red = increased expression in *Suv39h* dko cells, blue = reduced expression in *Suv39h* dko cells). Selected LINE families are labeled.

(f) Bisulfite-PCR analysis for DNA methylation in LINE1 regions. Plots display the percentages of DNA methylation in individual CpG positions of LINE1 PCR fragments. DNA methylation analysis was performed in control and *Suv39h* dko ESCs and DE cells. Data are presented as mean values +/- SD from replicate experiments (n=2; 500 sequences each).

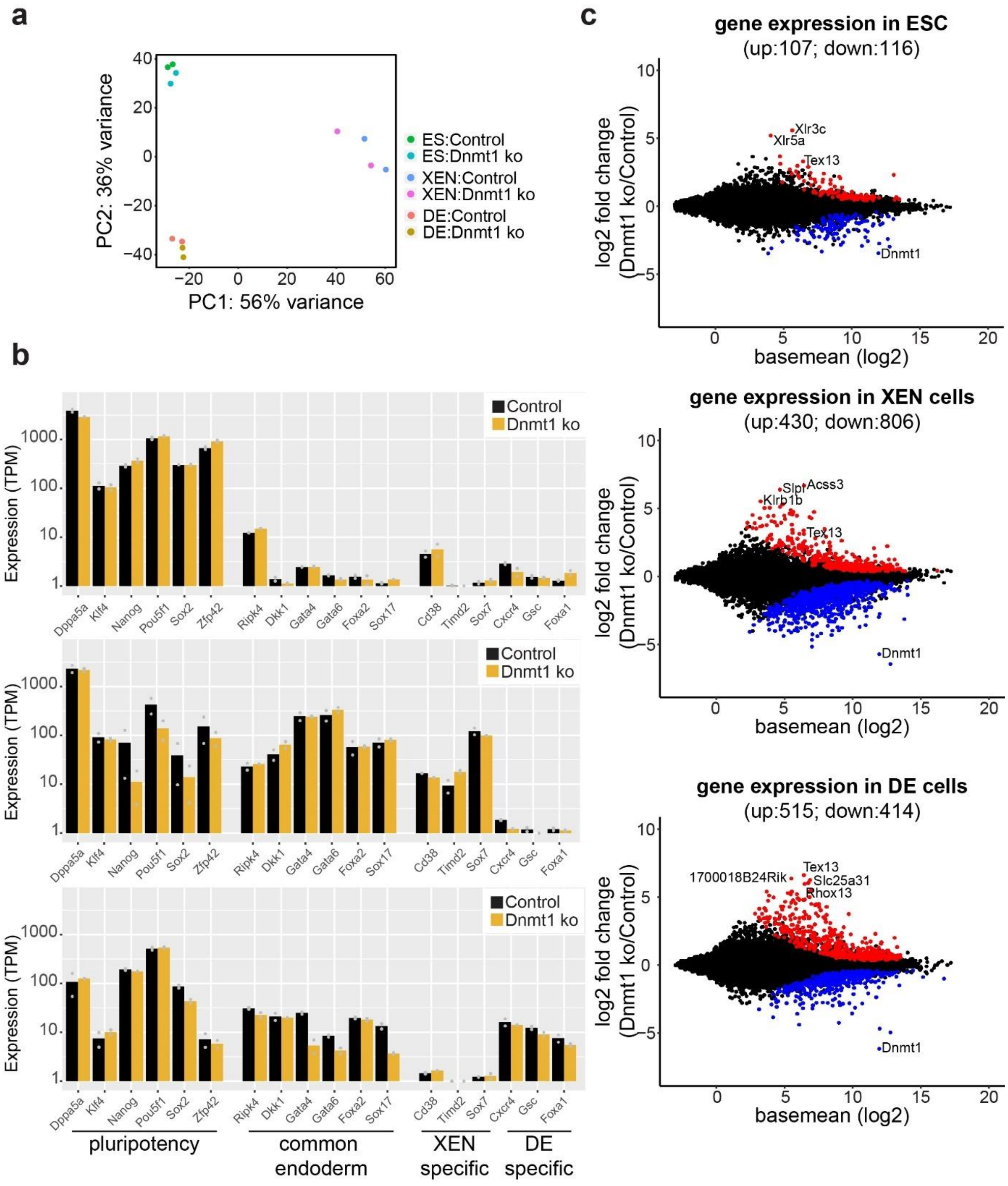
a
PC1: 56% variance
PC2: 36% variance
ES:Control
ES:Dnmt1 ko
XEN:Control
XEN:Dnmt1 ko
DE:Control
DE:Dnmt1 ko
b
Control
Dnmt1 ko
Expression (TPM)
Dppa5a
Klf4
Nanog
Pou5f1
Sox2
Zfp42
Ripk4
Dkk1
Gata4
Gata6
Foxa2
Sox17
Cd38
Timd2
Sox7
Cxcr4
Gsc
Foxa1
pluripotency
common endoderm
XEN specific
DE specific
c
gene expression in ESC
(up:107; down:116)
gene expression in XEN cells
(up:430; down:806)
gene expression in DE cells
(up:515; down:414)
log2 fold change (Dnmt1 ko/Control)
basemean (log2)
Xlr3c
Xlr5a
Tex13
Dnmt1
Slpi
Acss3
Klrb1b
1700018B24Rik
Slc25a31
Rhox13

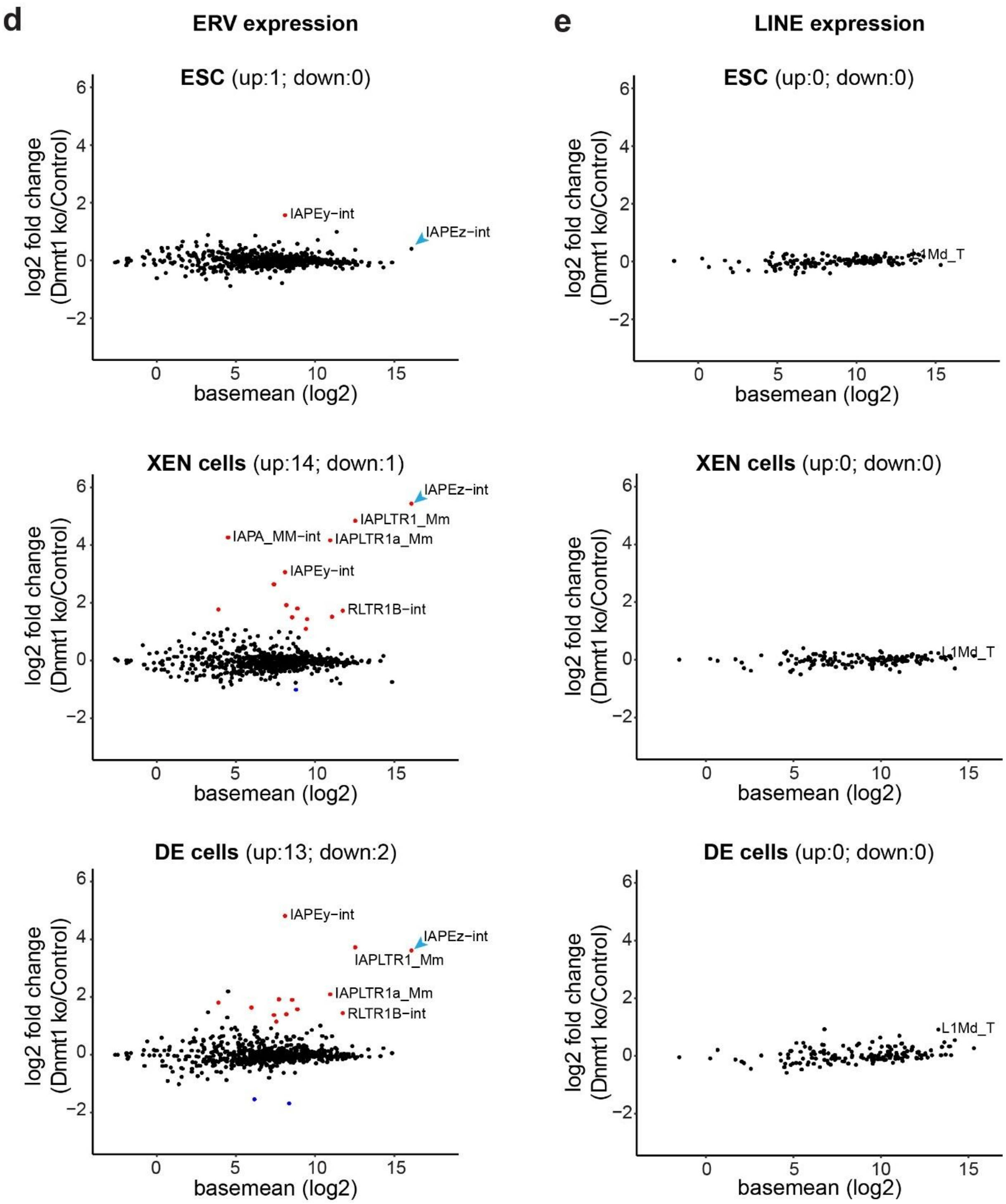
d
ERV expression
ESC (up:1; down:0)
IAPEy−int
IAPEz−int
XEN cells (up:14; down:1)
IAPEz−int
IAPLTR1_Mm
IAPA_MM−int
IAPLTR1a_Mm
IAPEy−int
RLTR1B−int
DE cells (up:13; down:2)
IAPEy−int
IAPEz−int
IAPLTR1_Mm
IAPLTR1a_Mm
RLTR1B−int
log2 fold change
(Dnmt1 ko/Control)
basemean (log2)
e
LINE expression
ESC (up:0; down:0)
L1Md_T
XEN cells (up:0; down:0)
L1Md_T
DE cells (up:0; down:0)
L1Md_T
log2 fold change
(Dnmt1 ko/Control)
basemean (log2)

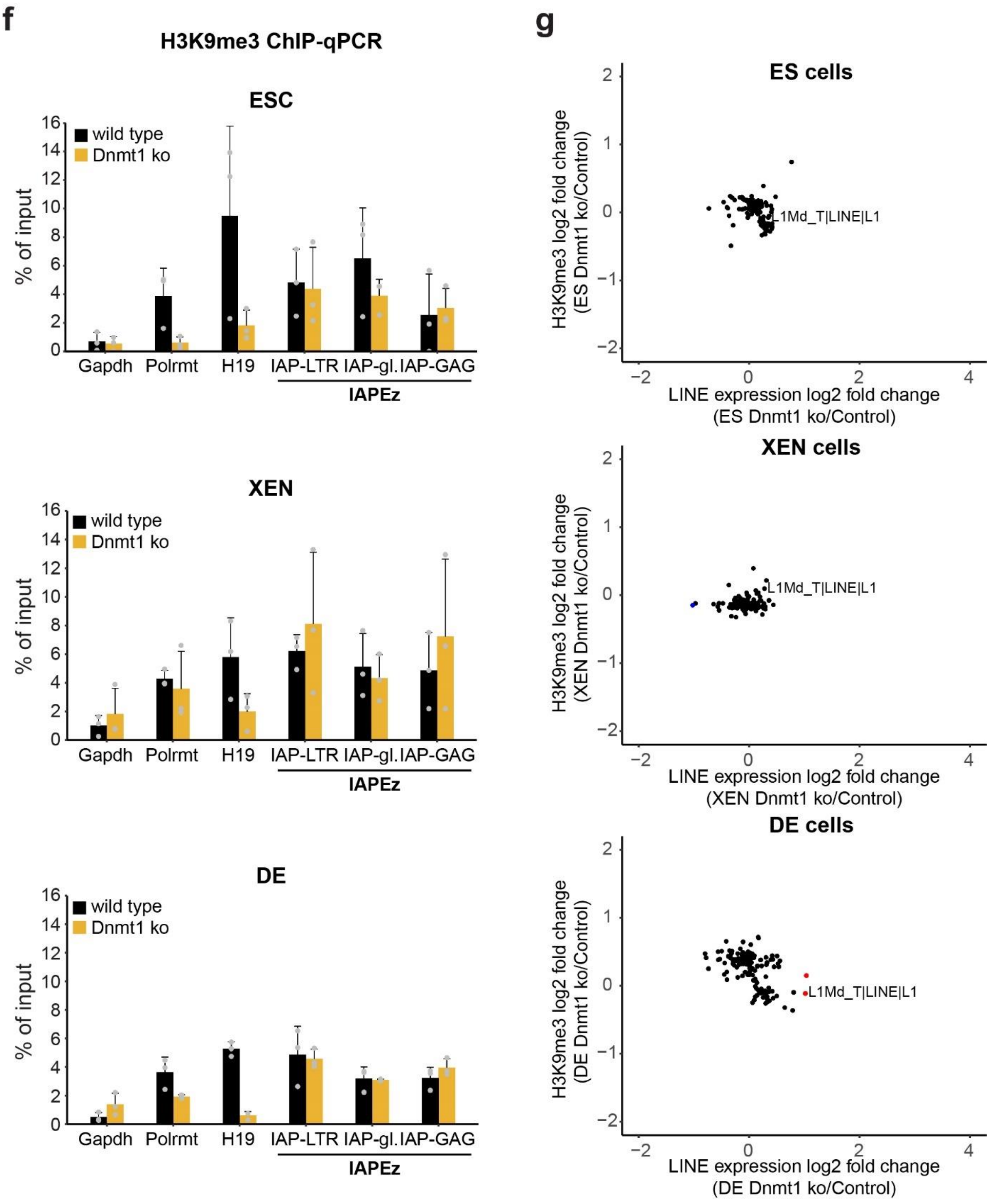

Supplementary Figure 9.

(a) PCA analysis of RNA-seq data from wild type and *Dnmt1* ko ESCs, XEN and DE cells. Each dot represents one individual replicate.

(b) Average expression levels of genes detected by RNA-seq for pluripotency, extra-embryonic endoderm and definitive endoderm markers in ESCs, XEN and DE cells. TPM: Transcripts Per Kilobase Million.

(c) Dot plot showing basemean expression vs. log2-fold change of protein coding genes in *in vitro* differentiated wild type vs. *Dnmt1* ko ESCs, XEN and DE cells. Genes with significantly changed expression (Wald test with Benjamini-Hochberg correction, adjusted p-value < 0.01; n=2 for each condition) are colored (red = increased expression in *Dnmt1* ko cells, blue = reduced expression in *Dnmt1* ko cells). Selected genes are labeled.
(d) Dot plot showing basemean expression vs. log2 fold change of ERV families in *in vitro* differentiated wild type and *Dnmt1* ko ESCs, XEN and DE cells. ERV families with significantly changed expression (Wald test with Benjamini-Hochberg correction, adjusted p-value < 0.01, fold change > 2; n=2 for each condition) are colored (red = increased expression in *Dnmt1* ko cells, blue = reduced expression in *Dnmt1* ko cells). Selected ERV families are labeled.
(e) Dot plot showing expression vs. H3K9me3 changes on LINEs between wild type and *Dnmt1* ko ESCs, XEN and DE cells. LINEs with significantly changed expression (fold change > 2; n=2 for each condition) are colored (red = increased expression in Dnmt1 ko cells). Selected LINEs are labeled.
(f) H3K9me3 is unaltered on IAP elements in *Dnmt1* ko cells. H3K9me3 ChIP-qPCR analysis in wild type (J1) and *Dnmt1* ko ESCs, XEN and DE cells. Bar plots indicate mean H3K9me3 enrichment over input. Data are presented as mean values +/- SD from replicate experiments (n=3). Positive control regions: *Polrmt*, *H19*; Negative control region: *Gapdh*. Source data are provided as a Source Data file.
(g) Dot plot showing expression vs. H3K9me3 changes between control and *Dnmt1* ko ESC, XEN and DE cells. LINEs with significantly changed expression (fold change > 2; n=2 for each condition) are colored (red = increased expression in *Dnmt1* ko cells). Selected LINEs are labeled.

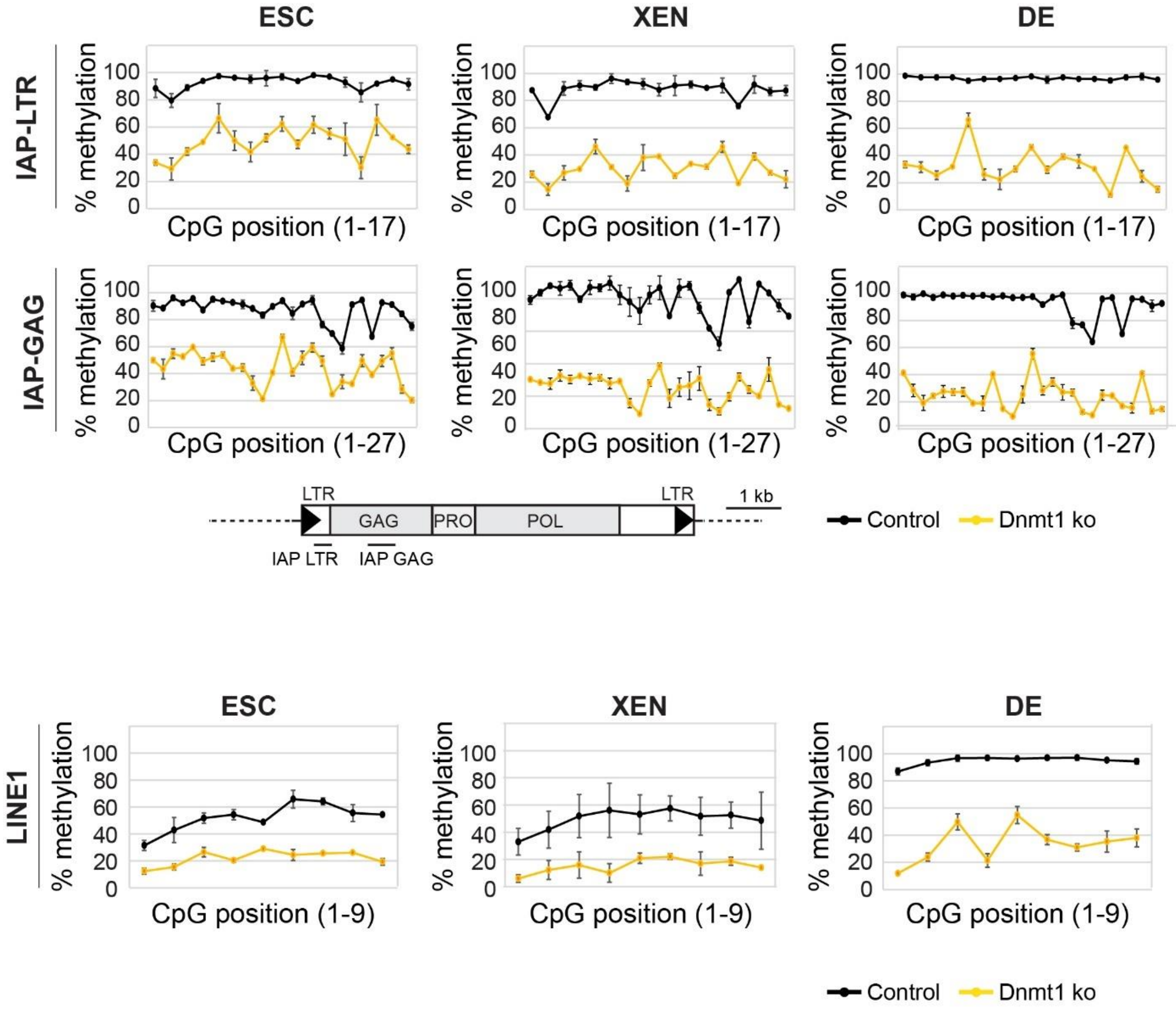

Supplementary Figure 10.

Bisulfite-PCR analysis for DNA methylation in *IAP-LTR*, *IAP-GAG* and LINE1 regions. Positions of the PCR products are indicated in the schematic. Plots display the percentages of DNA methylation in individual CpG positions of *IAP-LTR*, *IAP-GAG* and LINE1 PCR fragments. DNA methylation analysis was performed in control and *Dnmt1* ko ESCs, XEN and DE cells. Data are presented as mean values +/- SD from replicate experiments (n=2; 500 sequences each). Source data are provided as a Source Data file.
