# Supplementary material for: Dominant role of DNA methylation over H3K9me3 for IAP silencing in endoderm
Source: Nat Commun. 2022 Sep 19;13:5447. doi: 10.1038/s41467-022-32978-7 (PMC9485127; doi:10.1038/s41467-022-32978-7)
Supplement: Supplementary file 3 — Description of additional Supplementary File [file 41467_2022_32978_MOESM3_ESM.pdf]

Descriptions of additional Supplementary Files

Supplementary Data 1. Significantly regulated genes between control and Setdb1END embryonic endoderm cells.

Supplementary Data 2. Significantly regulated ERVs between control and Setdb1END embryonic endoderm cells.

Supplementary Data 3. Significantly regulated genes between in vitro differentiated control and Setdb1END XEN cells.

Supplementary Data 4. Significantly regulated genes between in vitro differentiated control and Setdb1END DE cells.

Supplementary Data 5. Significantly regulated ERVs between in vitro differentiated control and Setdb1END XEN cells.

Supplementary Data 6. Significantly regulated ERVs between in vitro differentiated control and Setdb1END DE cells.

Supplementary Data 7. Significantly regulated genes between control and Dnmt1 ko ES cells.
Supplementary Data 8. Significantly regulated genes between in vitro differentiated control and Dnmt1 ko DE cells.

Supplementary Data 9. Significantly regulated genes between in vitro differentiated control and Dnmt1 ko XEN cells.

Supplementary Data 10. Significantly regulated ERVs between control and Dnmt1 ko ES cells.

Supplementary Data 11. Significantly regulated ERVs between in vitro differentiated control and Dnmt1 ko DE cells.

Supplementary Data 12. Significantly regulated ERVs between in vitro differentiated control and Dnmt1 ko XEN cells.

Supplementary Data 13. Next generation sequencing datasets generated and analyzed in this study.
